# Measurements of Defect Structures by Positron Annihilation Lifetime Spectroscopy of the Tellurite Glass 70TeO_2_-5XO-10P_2_O_5_-10ZnO-5PbF_2_ (X = Mg, Bi_2_, Ti) Doped with Ions of the Rare Earth Element Er^3+^

**DOI:** 10.1186/s11671-017-2075-z

**Published:** 2017-04-26

**Authors:** K. Pach, J. Filipecki, E. Golis, El. S. Yousef, V. Boyko

**Affiliations:** 1 Institute of Physics, Faculty of Mathematics and Natural Science, Jan Dlugosz University in Czestochowa, Al. Armii Krajowej 13/15, 42-200 Czestochowa, Poland; 2 Physics Dep., Faculty of Science, Al. - Azhar University, Assiut Branch, Assiut, Egypt; 3 Institute of Physical Optics, Dragomanov str., 23, 79005 Lviv, Ukraine

## Abstract

The objective of the study was the structural analysis of the 70TeO_2_-5XO-10P_2_O_5_-10ZnO-5PbF_2_ (X = Mg, Bi_2_, Ti) tellurite glasses doped with ions of the rare-earth elements Er^3+^, based on the PALS (positron annihilation lifetime spectroscopy) method of measuring positron lifetimes. Values of positron lifetimes and the corresponding intensities may be connected with the sizes and number of structural defects, the sizes of which range from a few angstroms to a few dozen nanometers. Experimental positron lifetime spectrum revealed existence of two positron lifetime components *τ*_1_ and*τ*_2_. Their interpretation was based on two-state positron trapping model where the physical parameters are the positron annihilation rate and positron trapping rate.

## Background

Tellurite glasses belong to a group of materials which, due to their properties, can be applied in optoelectronics and photonics, as fast ion conductors, photonic materials and in lasers [1, 2]. Tellurite glasses are treated as special glasses on account of the separately molten TeO_2_, which does not display glass-making properties. If TeO_2_ is connected with a stabilizing oxide, the glass-making properties of the tellurite oxide are determined. Due to the fact that the tellurite glasses are characterised by high linear as well as nonlinear index, they have become the major research topic of nonlinear optics [3, 4]. High density of tellurite glasses, their low glassy state transition temperature and wide range of IR permeability (melting point lower than 1000 °C) [1] should also be noted.

Tellurite glasses are characterized by photon energy which is 750 cm^−1^, thus determining the probability of radiative transitions as well as longer lifetime levels of energetic ions of the rare earth elements [5]. Low energy of the photons of the tellurite glasses allows to use them as materials for building optical amplifiers and laser devices. An important characteristic of tellurite glasses is their ease of dissolving rare earth elements, such as Pr^3+^, Er^3+^, Nd^3+^, etc. An inseparable element of this phenomenon is the increase of the luminescence effects required in laser glasses [6, 7]. Due to their physic-chemical properties, there is a need for further research on the glasses. Changing the chemical constitution of the glasses as well as different methods of synthesis make possible a wide range of their formation in terms of optical and technological properties [8–11].

It is certainly true that one of the most important properties of tellurite glasses is their ability to attach ions of the rare earth elements, which results in an increased effectiveness of the luminescence, which is required in laser glasses. Unique properties of the tellurite glasses cause that there is a very wide potential for their application. Application of tellurite glasses are results from the atomic structure of the material, as well as from the structure of the voids (gaps, free volumes). The structure of telluric glasses have areas with different degrees of order in the form of a coordination polyhedrons forming the roof construction: glass and having their crystalline counterparts, and empty spaces in which ions may be glass modifiers [12, 13]. To describe the actual structure of glass and nanocrystalline glass-ceramics is necessary to develop alongside conventional methods such as X-ray diffraction, electron or neutron diffraction of high energy synchrotron radiation Raman spectroscopy, infrared spectroscopy, the new experimental methods sensitive to the voids in the structure of glasses. This idea can be realized thanks to the free annihilation of particle with an anti-particle, with a simultaneous emission of two gamma quanta (2γ). In such cases, two positron lifetime components *τ*_1_ and *τ*_2_ are obtained during measurements of positron lifetime spectroscopy PALS [14–17]. The *τ*_1_ component is responsible for positron and electron free annihilation, as well as for the annihilation with electrons localized in point defects of the vacancy type, whereas the *τ*_2_ component is connected with the electrons of volume anionic defects created in mono-vacancies as well as in defects formed on grain boundaries or dislocations [18–20]. Mathematical formalism of the known two-state positron trapping model with only one kind of traps [21] was utilized to parameterize mean *τ*_av_ and defect-free bulk *τ*_b_ lifetimes, as well as trapping grate in defects *κ*_d_. In addition:

1. average positron lifetime τ_av_ reflecting defectivity of the medium dominating in the examined glasses:
  1 $$ {\tau}_{av=}\frac{\tau_1{I}_1+{\tau}_2{I}_2}{I_1+{I}_2} $$
2. bulk positron lifetime *τ*_*b*_ in the defect-free part of the material:
  2 $$ {\tau}_b=\frac{I_1+{I}_2}{\frac{I_1}{\tau_1}+\frac{I_2}{\tau_2}} $$
3. positron capture rate by the traps (defects) *κ*_d_:
  3 $$ {K}_d=\frac{I_2}{I_1}\left(\frac{1}{\tau_b}-\frac{1}{\tau_2}\right) $$
4. a quantity connected with the average size of defects in which annihilation takes place:
  4 $$ {\tau}_2-{\tau}_b $$
5. the parameter change reflects the change in the geometry of defects in volume, nature of capture and positron trapping centres [22]:
  5 $$ {\tau}_2/{\tau}_b $$
6. trapped positron fraction *η*:
  6 $$ \eta ={\tau}_1{\kappa}_d $$

## Experiment and methods

**Fig. 1:**
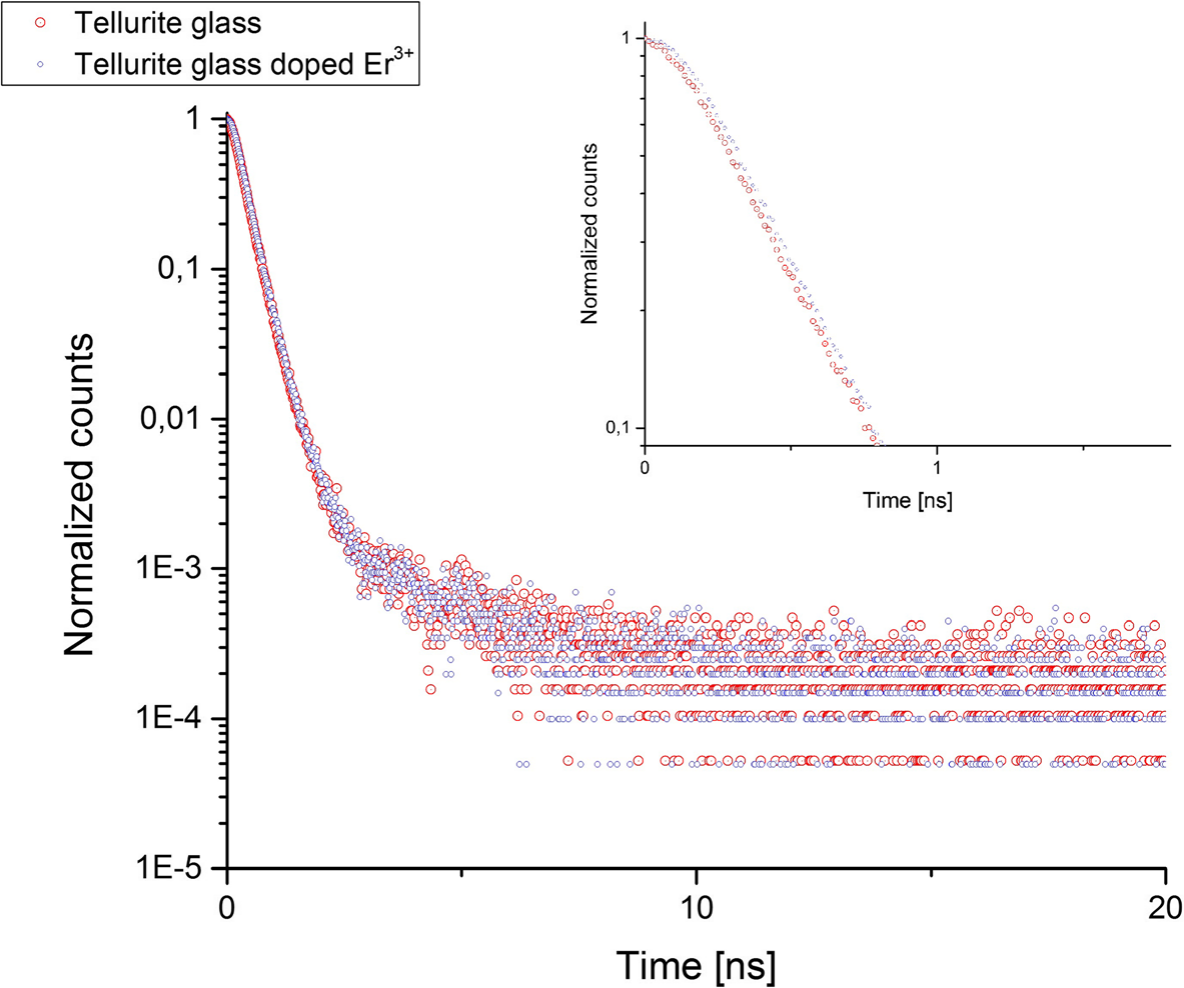
The examples of experimental PALS spectra of tellurite glasses and tellurite glasses doped Er^3+^ samples

Tellurite glasses were melted in an electric furnace at the temperature of 850 °C in the air atmosphere in platinum-gold melting pots with lids. Next, the melted material was poured into a brass form at the temperature of 380 °C. In order to eliminate the stresses created in the material, the glasses were subjected to relief annealing at the temperature of 380 °C for 2 h. Before the measurements were started, the tellurite glasses were subjected to grinding and polishing. The tellurite glasses, the composition of which was presented in Table 1, were examined. Positron annihilation lifetime spectroscopy PALS and spectrum analyses carried out with the use of the LT9 software were applied to examine parameters of tellurite glasses structural defects [23]. Measurements of positron lifetimes were made at room temperature with the use of ORTEC spectrometer [24, 25] based on the start-stop principle. Peak resolution FWHM of the apparatus was determined with the use of a ^60^Co radioactive source and was equal to 260 ps. The source of positrons was the ^22^Na sodium isotope of 4 × l0^−5^ Bq activity closed in kapton film. Together with the samples, it formed a “sandwich” type system. The measurements were carried out at room temperature. The examples of experimental PALS spectra of tellurite glasses and tellurite glasses doped Er^3+^ samples are shown in Fig. 1.

**Table 1:** Chemical composition of tellurite glasses

Sample	Composition [%mol]
Te 1	70TeO_2_-5MgO-10P_2_O_5_-10ZnO-5PbF_2_
Te 2	70TeO_2_-5MgO-10P_2_O_5_-10ZnO-5PbF_2_ + 600[ppm]Er_2_O_3_
Te 3	70TeO_2_-5Bi_2_O-10P_2_O_5_-10ZnO-5PbF_2_
Te 4	70TeO_2_-5Bi_2_O-10P_2_O_5_-10ZnO-5PbF_2_ + 600[ppm]Er_2_O_3_
Te 5	70TeO_2_-5TiO-10P_2_O_5_-10ZnO-5PbF_2_
Te 6	70TeO_2_-5TiO-10P_2_O_5_-10ZnO-5PbF_2_ + 600[ppm]Er_2_O_3_

## Results

The best fitting in tellurite glass samples was found to occur at the resolution of the annihilation spectrum into two components of *τ*_1_ and *τ*_2_ lifetimes and respective I_1_ and I_2_intensities (Table 2). No *τ*_3_ component, responsible for formation of the positronium (hydrogen-like atom) has been found in the examined samples. When analysing values of *τ*_1_ and *τ*_2_, the double-state model of positron annihilation was investigated, according to which a positron annihilates from a free state and from one of the states localized in a defect at the absence of the de-trapping process. Having calculated the main annihilation parameters with the use of the LT programme, positron lifetimes τ_1_ and τ_2_ and their intensity, as well as positron capture parameters *τ*_av_, *τ*_b_, *κ*_d_, *τ*_2_-*τ*_b_, *τ*_2_/*τ*_b,_ *η* (Table 3) were calculated. The main parameters of annihilation converted as mean ± measurement error were calculated using the program LT. Size was calculated from the average measurements of positron lifetime and intensity [23].

**Table 2:** Fitting parameters of tellurite glasses mathematically treated with two-component fitting procedure by LT computer program

Sample	*τ*_1_[ns]	*I*_1_ [%]	*τ*_2_ [ns]	*I*_2_ [%]
Te 1	0.181 ± 0.002	75.31 ± 0.51	0.374 ± 0.005	24.69 ± 0.74
Te 2	0.181 ± 0.002	71.63 ± 0.84	0.379 ± 0.006	28.37 ± 0.63
Te 3	0.176 ± 0.003	72.32 ± 0.92	0.353 ± 0.007	27.68 ± 0.82
Te 4	0.171 ± 0.003	67.27 ± 0.83	0.332 ± 0.004	32.73 ± 0.84
Te 5	0.179 ± 0.003	70.64 ± 0.83	0.360 ± 0.006	29.36 ± 0.83
Te 6	0.171 ± 0.003	66.03 ± 0.92	0.342 ± 0.006	33.97 ± 0.85

**Table 3:** Positron capture parameters

Sample	*τ*_av_[ns]	*τ*_b_[ns]	*κ*_d_[ns^-1^]	*τ*_2_-*τ*_b_[ns]	*τ*_2_/*τ*_b_	*η*[au]
Te 1	0.229 ±0.005	0.207 ±0.004	0.704 ±0.013	0.167 ±0.003	1.803 ±0.041	0.127 ±0.002
Te 2	0.237 ±0.005	0.212 ±0.004	0.819 ±0.022	0.167 ±0.003	1.784 ±0.042	0.148 ±0.003
Te 3	0.225 ±0.005	0.204 ±0.004	0.789 ±0.022	0.149 ±0.003	1.727 ±0.031	0.138 ±0.003
Te 4	0.224 ±0.004	0.203 ±0.004	0.928 ±0.021	0.129 ±0.002	1.633 ±0.033	0.158 ±0.003
Te 5	0.232 ±0.005	0.210 ±0.004	0.825 ±0.021	0.150 ±0.003	1.714 ±0.032	0.147 ±0.003
Te 6	0.229 ±0.005	0.206 ±0.004	0.993 ±0.021	0.136 ±0.003	1.662 ±0.032	0.169 ±0.003

## Discussion

On the basis of the taken measurements of positron lifetimes, *τ*_1_ and *τ*_2_ are slightly different, within the margin of errors, for the basic samples and for the doped samples. Distinct changes occur in case of values of the *I*_1_ and *I*_2_intensities. After doping the basic glass with Er_2_O_3_ admixture, a significant increase of the *I*_2_ intensity can be observed. In the examined glasses, the number of volume defects increases together with addition of ions of the rare earth elements.

Basing on the taken measurements, it is possible to state that doping the basic sample with the Er_2_O_3_ ions causes distinct changes in the values of parameters responsible for positron capture. It can be concluded that the capture of positrons are responsible anionic gaps in the form of voids or defects in the structure.

Taking into account calculations of positron capture parameters (Table 2), it is possible to state that:

- ➢Rates of the *κ*_d_ positron capture, when compared to the basic samples, distinctly increase during doping in all the investigated tellurite glasses. This reflects a much higher concentration of volume defects and positron capture centres in the samples doped with Er_2_O_3_ ions.
- ➢The value of the *τ*_2_-*τ*_b_ parameter for the Te2 sample remains at the same level, whereas it distinctly falls for the other samples of the investigated tellurite glasses. It means that in the other samples average sizes of defects, in which positron captures occur, decrease during doping.
- ➢The *τ*_2_/*τ*_b_ ratio diminishes during doping of the basic sample, which indicates different nature of volume defects. Places in which positrons are captured are of different nature, depending on embedding erbium oxide in the structure of a glass in the examined materials.
- ➢The *η* fraction of the trapped positrons distinctly increases in the investigated samples when the basic sample is doped with the erbium ions, where the value increases by c. 15%, which is evidence of increasing free volume fraction.

## Conclusions

To sum up, analysis of the examined tellurite glasses according to the double-state model, for the *τ*_av_, *τ*_b_, *κ*_d_, *τ*_2_-*τ*_b_, *τ*_2_/*τ*_b,_ and *η* parameters confirms division of the examined samples into two groups: clean and doped with the Er_2_O_3_ ions. Three parameters *τ*_av_, *τ*_b_,*τ*_2_/*τ*_b_ for the samples Te1, Te2 do not demonstrate any distinct division (changes occur within the margin of error), parameters are not changed or are maintained at the same level under the influence of doping. As for the other samples of the examined tellurite glasses both, the nature of volume defects and average size of defects undergo reduction. The changes may result from the properties of an agent that can be found in each of the examined samples. In the samples Te1, Te2 the agent is the oxide which shows paramagnetic properties, whereas in the other samples, it is the oxide which displays diamagnetic properties. The rate of positron capture by the traps increases in all the examined samples and so does the fraction of the trapped positrons.

## Abbreviations

FWHM: Full width at half maximum
LT9: Lifetime programme
PALS: Positron annihilation lifetime spectroscopy
